# Optimization of Giant Unilamellar Vesicle Electroformation for Phosphatidylcholine/Sphingomyelin/Cholesterol Ternary Mixtures

**DOI:** 10.3390/membranes12050525

**Published:** 2022-05-16

**Authors:** Zvonimir Boban, Ivan Mardešić, Witold Karol Subczynski, Dražan Jozić, Marija Raguz

**Affiliations:** 1 Department of Medical Physics and Biophysics, University of Split School of Medicine, 21000 Split, Croatia; zvonimir.boban@mefst.hr (Z.B.); imardesi@mefst.hr (I.M.); 2 Faculty of Science, University of Split, Doctoral Study of Biophysics, 21000 Split, Croatia; 3 Department of Biophysics, Medical College of Wisconsin, Milwaukee, WI 53226, USA; subczyn@mcw.edu; 4 Faculty of Chemistry and Technology, University of Split, 21000 Split, Croatia; jozicd@ktf-split.hr

**Keywords:** GUVs, electroformation, cholesterol, sphingomyelin, phosphatidylcholine, film thickness, frequency, voltage, AFM

## Abstract

Artificial vesicles are important tools in membrane research because they enable studying membrane properties in controlled conditions. Giant unilamellar vesicles (GUVs) are specially interesting due to their similarity in size to eukaryotic cells. We focus on optimization of GUV production from phosphatidylcholine/sphingomyelin/cholesterol mixtures using the electroformation method. This mixture has been extensively researched lately due to its relevance for the formation of lipid rafts. We measured the effect of voltage, frequency, lipid film thickness, and cholesterol (Chol) concentration on electroformation successfulness using spin-coating for reproducible lipid film deposition. Special attention is given to the effect of Chol concentrations above the phospholipid bilayer saturation threshold. Such high concentrations are of interest to groups studying the role of Chol in the fiber cell plasma membranes of the eye lens or development of atherosclerosis. Utilizing atomic force and fluorescence microscopy, we found the optimal lipid film thickness to be around 30 nm, and the best frequency–voltage combinations in the range of 2–6 V and 10–100 Hz. Increasing the Chol content, we observed a decrease in GUV yield and size. However, the effect was much less pronounced when the optimal lipid film thickness was used. The results underline the need for simultaneous optimization of both electrical parameters and thickness in order to produce high-quality GUVs for experimental research.

## 1. Introduction

Membrane bilayers are crucial for the proper functioning of biological cells. However, due to their complexity, it is often hard to evaluate a specific property while controlling for all other properties that also might influence the measurement. This is why many studies concerned with specific membrane properties use artificial vesicles. Giant unilamellar vesicles (GUVs) are of special importance since their size in the range of 5–100 μm is comparable to the size of eukaryotic cells. Additionally, this size range is optimal for studies utilizing light microscopy.

**Figure 1:**
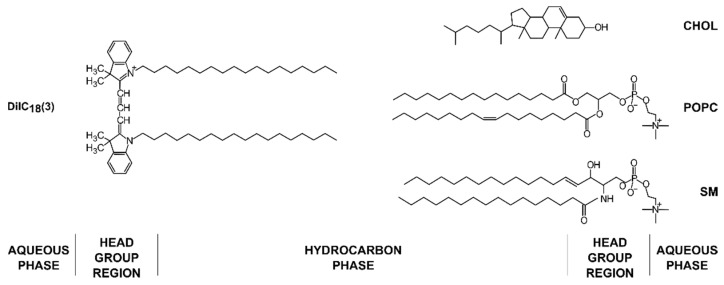
Chemical structures of lipids and the fluorescent dye used in the experiments with their approximate locations in the membrane bilayer.

The most commonly used method for growth of GUVs is electroformation. It was developed by Angelova and Dimitrov in 1986 [1] and has constantly been perfected and updated since. The method consists of depositing a lipid mixture of choice on a conductive substrate. The dried film is then hydrated, and an alternating current applied to detach the lipid film from the surface and form GUVs. Although quite simple at first glance, the method is influenced by many parameters, requiring optimization depending on the experimental setup [2]. We focus on the effect of lipid composition, lipid film thickness, and electrical parameters (frequency and voltage) using a ternary mixture of 1-palmitoyl-2-oleoyl-glycero-3-phosphocholine/sphingomyelin/cholesterol (POPC/SM/Chol) (Figure 1). This mixture was chosen since these three type of lipids are the most abundant in biological membranes [3,4,5]. Of special interest currently is the state of liquid order–liquid disorder (Lo–Ld) phase separation which is important for the formation of lipid rafts [6]. Although substantial research has been conducted using GUVs of such compositions, we are aware of no study systematically dealing with optimization of the manufacturing process for those compositions using electroformation.

In addition to frequency and voltage, lipid film thickness is an important electroformation parameter whose importance is often overlooked. Some studies dealing with electroformation optimization focused solely on optimizing the electrical parameters. This limits the usefulness of the results, since lipid film thickness inevitably affects the electric field and consequent membrane fluctuation [7,8]. Consequently, using different lipid thicknesses with the same electrical parameters should provide different results. Additionally, the traditional electroformation protocol uses drop deposition in which the lipids are simply dropped on the surface and the solvent is evaporated afterward. This leaves a lipid film of uneven thickness, reducing the homogeneity of obtained GUVs and reproducibility of the experiment. Several methods have been suggested for achieving reproducible lipid film thicknesses [9,10,11]. Here, we use the spin-coating method in which lipids are deposited onto the electrode and then spun at a high angular velocity in order to obtain a homogeneous lipid film for electroformation [9].

The effect of different Chol concentrations, including those exceeding its membrane saturation limits, is also explored. Such high Chol concentrations are interesting to researchers investigating the role of Chol in fiber cell plasma membranes of the eye lens [12,13,14,15,16,17] or the development of atherosclerosis [18,19]. Within the human eye lens, the Chol/phospholipid (Chol/PL) molar ratio is in the range of 1–2 in the lens cortex and 3–4 in the lens nucleus [20,21]. Such high Chol contents are thought to be crucial for maintaining lens transparency by enabling the formation of Chol bilayer domains (CBDs) which ensure that the surrounding phospholipid bilayer is saturated with Chol.

Applying traditional electroformation protocols for production of GUVs from mixtures containing large quantities of Chol leads to Chol demixing artefacts and formation of Chol crystals [13,22,23]. These issues were encountered when we tried to confirm the existence of CBDs in Chol/POPC GUVs using confocal microscopy [13]. We were able to observe CBDs, but only when the Chol/POPC mixing ratio was equal to or greater than 3. This was in contrast with our earlier studies showing that CBDs start to appear at a Chol/PL ratio of 1/1 (50 mol.% Chol) in multilamellar vesicles [15,24]. However, when producing multilamellar vesicles, the rapid solvent exchange method can be used [25,26], which has been shown to be effective at protecting against the Chol demixing artefact. This confirms the significant effect of Chol demixing during the drying phase of the protocol, resulting in different lipid ratios inside the GUV membranes (molar ratios) compared to the initial ratios in the lipid mixture (mixing ratios). Consequently, in order to cover the Chol concentrations needed for CBD formation, we used mixing ratios of Chol/(POPC + SM) in the range of 0–3.5.

## 2. Materials and Methods

### 2.1. Materials

POPC, egg SM, and Chol were obtained from Avanti Polar Lipids Inc. (Alabaster, AL, USA). The fluorescent dye 1,1′-dioctade-cyl-3,3,3′,3′-tetramethylindocarbocyanine Perchlorate (DiIC_18_(3)) (Figure 1) was purchased from Invitrogen, Thermo Fisher Scientific (Waltham, MA, USA). When not used, the lipids were stored at −20 °C. Other chemicals of at least reagent grade were obtained from Sigma-Aldrich (St. Louis, MO, USA). Indium tin oxide-coated glass (ITO, CG-90 INS 115) was purchased from Delta Technologies (Loveland, CO, USA). ITO glass dimensions were 25 mm × 75 mm × 1.1 mm. New ITO glass was used for each preparation in order to prevent coating deterioration [27]. Mili-Q deionized water was used as the internal chamber solution.

### 2.2. Deposition of the Lipid Film

The spin-coating method was used for lipid film deposition [9]. Prior to spin-coating, the glass was immersed in deionized water for at least 45 min before being wiped four times with 70% ethanol moistened lint-free wipes. Properties of samples with Chol/(POPC + SM) mixing ratios in the range of 0–3.5 were compared (0–77.8 mol.% Chol in the mixture). The POPC/DiIC_18_(3) molar ratio was always kept at 1/0.002. The POPC/SM mixing ratio is fixed at 1/1. Lipid mixtures were prepared in 95% chloroform and 5% acetonitrile solution [9]. The solution (350 µL) was deposited onto the ITO surface, and a thin lipid layer was created using a Sawatec SM-150 spin-coater (Sawatec, Sax, Switzerland). The glass was spun for 4 min at 600 rpm with the final velocity reached in 1 s. After coating, the lipid film was placed under vacuum for 30 min to evaporate any remaining solvent.

### 2.3. Electroformation Chamber

**Figure 2:**
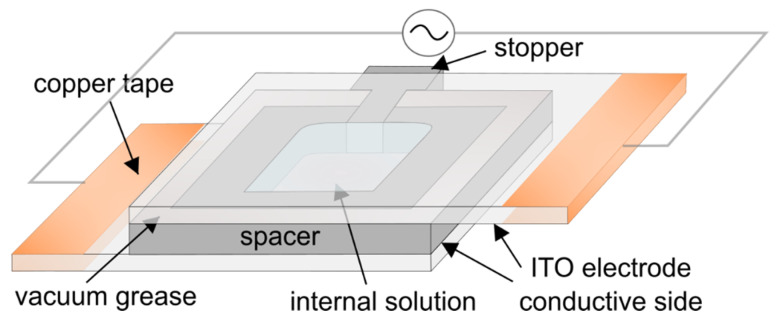
Schematic representation of the electroformation chamber used in the experiments.

The electroformation chamber was made of two 25 mm × 37.5 mm ITO-coated glass electrodes separated by a 1.6 mm thick Teflon spacer (Figure 2). The electrodes were made by cutting a 25 mm × 75 mm ITO glass slide in half using a diamond pen cutter. After spin-coating lipids on one of the electrodes, the chamber was assembled by attaching the spacer to the electrodes using vacuum grease. Upon insertion, the stopper was also sealed with vacuum grease. In this way, contact between the grease and the internal solution was avoided, minimizing the possibility of harmful effects due to grease contamination [28]. Finally, the chamber was attached to a voltage source (UNI-T UTG9005C, Chengdu, China pulse generator or a Joy-IT PSG 9080, Neukirchen-Vluyn, Germany signal generator) and placed inside an incubator at a temperature of 60 °C. In order to assure good contact between the conductor wires and the electrodes, the outer edges of the electrodes were covered with copper tape. After 2 h, the voltage was turned off and the chamber was kept in an incubator for another hour. When examining the effect of Chol concentration on average GUV diameter, electroformation was performed simultaneously on six samples by connecting the chambers in parallel.

### 2.4. Thickness Measurements

Atomic force microscopy (AFM) images were obtained using a JPK NanoWizard 4 system (JPK/Bruker, Berlin, Germany). Imaging was carried out in tapping mode. The image resolution was 512 pixels per line. Analysis was performed using the Gwyddion 2.60 software. In order to obtain the thicknesses, a cut was made on the lipid film deposited on ITO glass. The depth of the cut was assessed by calculating the height difference between the bottom of the trough and the height of the plateau next to the cut (far away enough from the edge of the cut ensuring that the measurements represent the unperturbed lipid film). Final thickness was an average obtained from measurements of five height profiles perpendicular to the cut. In order to confirm the flatness of the ITO layer prior to film deposition, we performed AFM measurements on clean ITO slides and found the surface roughness to be in the range of couple nanometers, in accordance with the results presented by Herold et al. [27].

The X-ray reflectivity (XRR) experiments were performed using an Empyrean X-ray diffraction system (Malvern PANalytical, Malvern, UK) with a programmable *xy*-platform and *z*-adjustment at the position of the sample holder. The instrument was equipped with a copper X-ray tube (CuKα = 1.541 Å), multicore optics iCore/dCore, and PIXcel3D detector with Medipix-3 technology. The X-ray beam was parallelized using iCore optics and XRR curves were recorded under the following operating conditions: 45 kV anode voltage and 40 mA anode current. Scans were made using an Omega-2Theta scan with a step size of 0.002° and a scanning angle in the range of −0.021° to 6°.

### 2.5. Fluorescence Imaging

In order to search the entire volume of the chamber, we collected images from 13 regions on the sample. If possible, up to 50 vesicles were tracked from each image. In order to reduce measurer’s bias, the computer randomly chose 30 vesicles out of these 50. If the image did not contain 30 vesicles, all of the tracked vesicles from that image were counted. Images were obtained using an Olympus BX51 (Olympus, Tokyo, Japan) fluorescence microscope. Vesicle diameters were measured using the line tool in Fiji software [29].

### 2.6. Data Analysis

If not stated otherwise, numerical results are expressed as the mean ± standard error. Sample distribution normality was tested using the Shapiro–Wilk test. Difference of means for two groups was tested using Student’s *t*-test. Goodness of linear fit was estimated using the coefficient of determination $R^{2}$. Data analysis and visualization were performed using the R programming language [30].

## 3. Results and Discussion

### 3.1. Effect of Electrical Parameters

In order to determine the optimal electrical parameters, we tested 25 frequency–voltage combinations with frequencies ranging from 1 to 1000 Hz and peak-to-peak voltages ranging from 0.5 to 20 V. A 3.75 mg/mL concentrated POPC/SM/Chol 1/1/1 mixture was used. Such a mixture should create GUVs in the Lo–Ld phase [31], which is important for studies of lipid rafts [6].

**Figure 3:**
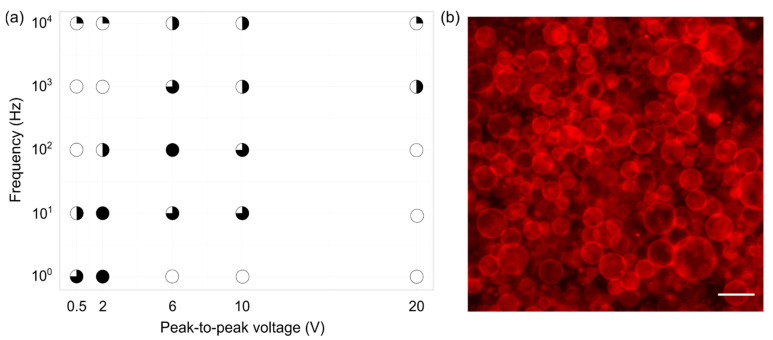
(**a**) Electroformation successfulness dependence on frequency–voltage combinations for a 1/1/1 POPC/SM/Chol mixture and a lipid concentration of 3.75 mg/mL. Successfulness takes into account the population homogeneity, yield, and size of GUVs, as well as the amount of defects. It is displayed here through circle fullness, where a fuller circle indicates better successfulness. Empty circles denote that no GUVs were formed or their number was negligible. (**b**) Fluorescence microscopy image of electroformed GUVs for a 10 Hz–2 V frequency–voltage combination. The scale bar in the bottom right corner denotes 50 μm.

Electroformation successfulness was estimated from fluorescence images, taking into account the GUV population homogeneity, yield, and size, as well as the amount of defects. It is denoted in Figure 3 by circles, where a fuller circle indicates better successfulness. Empty circles represent conditions for which GUV formation did not occur or was sporadic. The results indicate that the best frequency–voltage combinations were in the range of 10–100 Hz and 2–6 V (Figure 3a). For these combinations, the GUVs yield was high with sizes up to ~50 μm (Figure 3b). GUVs formation was observed for other combinations as well, but the successfulness was lower. It can also be noted that, in order to grow GUVs at higher voltages, higher frequencies were required. Such behavior can be explained through the effect of electrical parameters on lipid film fluctuations and lipid oxidation. Too strong electric fields can induce lipid oxidation [32,33] and tear the lipid film off the surface prematurely [34], negatively affecting GUV formation. Lipid film oscillations are important because they are responsible for lipid film bending and separation. These oscillations have been shown to rise with increasing electric fields and decrease with increasing frequencies [7,8,35]. This could explain why an increase in frequency is required in order to create GUVs at higher voltages. Increasing the frequency simultaneously with voltage keeps the oscillations in the optimal interval, ensuring that membrane fluctuations appear, but preventing an overly fast bilayer detachment. Furthermore, decreasing the voltage too much or overly increasing the frequency leads to too small membrane fluctuations, preventing the swelling required for GUV formation [36]. POPC and SM are monounsaturated lipids, and it has been shown that such lipids are not affected strongly by voltage-induced oxidation [33,37]. Consequently, the dominant effect here is probably related to oscillations intensity.

### 3.2. Effect of Lipid Film Thickness

Along with voltage and frequency, lipid film thickness is also a crucial electroformation parameter, but studies optimizing lipid thickness are scarce. The reason is probably mostly historical, since researchers often use the traditional drop-deposition protocol, in which film thickness can only be crudely estimated from the mass of deposited lipids and the deposition area. Furthermore, both measuring the thickness and using alternative protocols which enable reproducible film deposition require special equipment, again making it harder to conduct such experiments. Here, we utilized spin-coating [9] in order to test the effect of lipid film thickness on GUV electroformation from a ternary POPC/SM/Chol mixture.

We spin-coated the lipid solutions using four different lipid concentrations: 0.5, 2, 3.75, and 6 mg/mL. Two Chol/(POPC + SM) mixing ratios of 0.5 and 2 were inspected, giving a total of eight different parameter combinations tested. Measurements were performed three times for each different combination.

**Figure 4:**
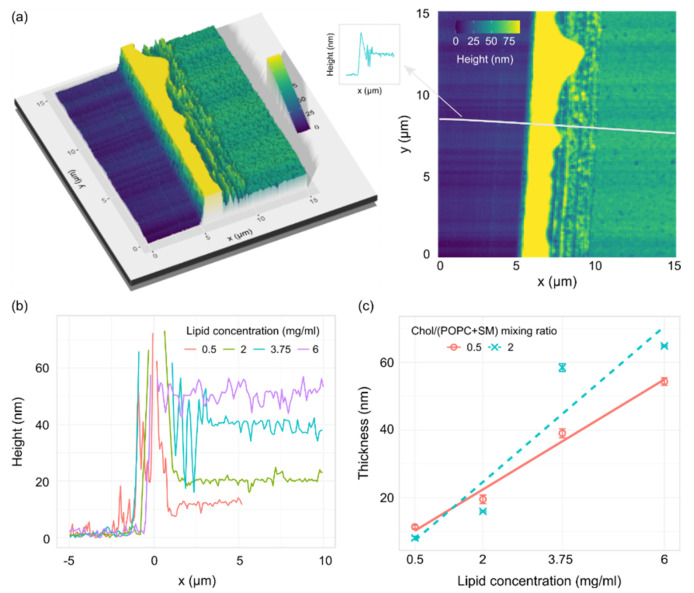
(**a**) 3D (left) and 2D (right) AFM images of a cut on a lipid film made from a 1/1/1 POPC/SM/Chol mixture with a lipid concentration of 3.75 mg/mL. The material buildup on the edge of the trough (yellow hill on the image) is cut off in order to better visualize the rest of the sample. The gray line on the 2D image shows the height profile position. The arrow points to a schematic representation of the height profile used for thickness measurements. (**b**) Representative AFM height profiles for a 1/1/1 POPC/SM/Chol mixture at different lipid concentrations. (**c**) Thickness of the lipid film for two POPC/SM/Chol mixtures depending on the concentration of lipids in the solution used for spin-coating. *R*^2^ = 0.98 and 0.83 for Chol/(POPC + SM) mixing ratios of 0.5 and 2, respectively.

The lipid surface was imaged using AFM, and thicknesses were determined from height profiles of the cut in the film (Figure 4a). The depth of the cut was assessed from the height profiles by calculating the difference between the bottom of the trough and the height of the plateau next to the cut (Figure 4b). The plateau measurements were taken far away enough from the edge of the cut so we could be certain that the measurements represent the unperturbed lipid film. Obtained thicknesses displayed a linear dependence on lipid concentration for both mixtures (Figure 4c). 

In addition to AFM, we performed XRR measurements for different lipid concentrations at the same Chol/(POPC + SM) mixing ratio (Figure S1). The measurements on uncoated ITO glass yielded thicknesses similar to those stated by the manufacturer (~25 nm) (Figure S1a). Using the XRR method on lipid films gave more than one value of thickness, which suggests some film inhomogeneity over larger scales. However, the results displayed a thickness trend corresponding to that obtained by AFM (Figure S1b).

**Figure 5:**
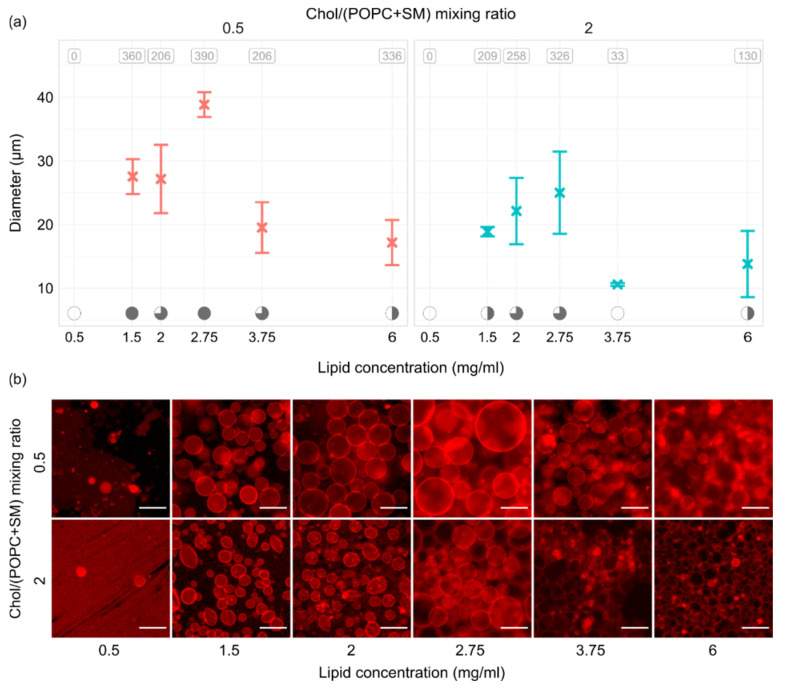
(**a**) GUV size depending on the thickness of the lipid film and Chol/(POPC + SM) mixing ratio. All of the experiments were performed using a frequency–voltage combination of 10 Hz–2 V. At the top of the panels, the average number of tracked vesicles per sample is displayed. Electroformation successfulness is displayed at the bottom of the panels. Successfulness takes into account the yield and size of GUVs and the amount of defects. It is displayed here through circle fullness, where a fuller circle indicates better successfulness. Empty circles denote that no GUVs were formed or electroformation efficiency was very low. (**b**) Representative fluorescence microscopy images for conditions displayed in the panels above. The scale bar in the bottom right of the images denotes 50 μm.

Figure 5 shows the results of experiments performed using six lipid concentrations and two different Chol/(POPC + SM) mixing ratios. Measurements were repeated three times for every combination. All of the experiments were performed using a frequency–voltage combination of 10 Hz–2 V. At the top of the panels in Figure 5a, the average number of tracked vesicles per sample is displayed. Electroformation successfulness is displayed at the bottom using the convention explained for Figure 3a. The trend was quite similar for both Chol/(POPC + SM) mixing ratios used. When we tested the lowest concentration of 0.5 mg/mL, practically no vesicles were observed due to a too small thickness of the film (~10 nm). The successfulness improved with increase in the lipid concentration, peaking at 2.75 mg/mL (film thickness ~30 nm) which seemed to be optimal for our mixtures. A decrease in successfulness then appeared as we moved on to the 3.75 and 6 mg/mL concentrations.

Observing Figure 5b, we can see that using the optimal lipid film thickness not only increases the yield and size of vesicles, but also contributes to a lower amount of defects in the sample. This was especially visible for the mixture with a higher concentration of Chol, where only the 2.75 mg/mL concentration seemed to provide the conditions for growth of dominantly defect-free vesicles.

### 3.3. Effect of Cholesterol Concentration

**Figure 6:**
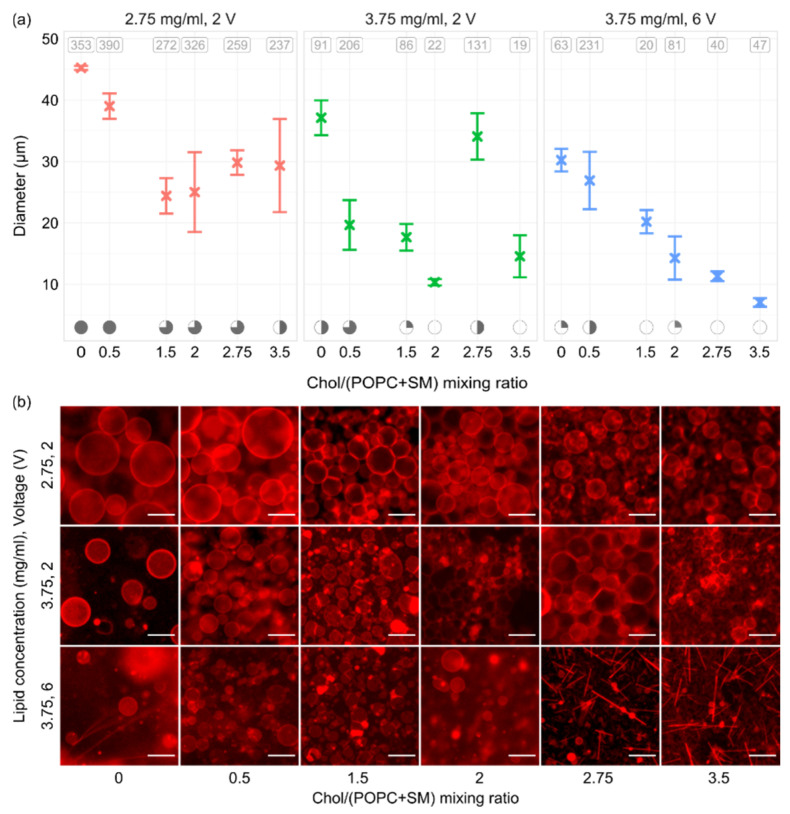
(**a**) GUVs sizes for different Chol concentrations, lipid concentrations (lipid film thicknesses), and voltages. All experiments were performed using a 1/1/1 POPC/SM/Chol mixture and a frequency of 10 Hz. At the top of the panels, the average number of tracked vesicles per sample is displayed. Electroformation successfulness is displayed at the bottom of the panels. Successfulness takes into account the yield and size of GUVs and the amount of defects. It is displayed here through circle fullness, where a fuller circle indicates better successfulness. Empty circles denote that no GUVs were formed or electroformation efficiency was very low. (**b**) Representative fluorescence microscopy images for conditions displayed in the panels above. The scale bar in the bottom right of the images denotes 50 μm.

In order to see how electroformation results changed with varying Chol content, we inspected Chol/(POPC + SM) mixing ratios in the range of 0–3.5 (0–77.8 mol.%). The Chol/(POPC + SM) mixing ratios were chosen so that, above the mixture transition temperature, GUVs with all possible bilayer phase domains could be grown (Ld, Lo–Ld, Lo, and CBDs). In order to assess the effects of both lipid film thickness and voltage, the experiments were performed for lipid concentrations of 2.75 and 3.75 mg/mL and voltages of 2 and 6 V. A frequency of 10 Hz was used for all experiments, and each parameter combination was tested three times. All three sets indicated a decrease in electroformation successfulness with increasing Chol content, with decreases in both GUV size and quality (Figure 6a,b). This is in agreement with our previous results using binary POPC/Chol mixtures [38], and it is probably due to large Chol concentrations increasing the rigidity of the lipid bilayer, causing more difficult bending during electroformation. Chol also has a condensing effect on the membrane, leading to smaller phospholipid surface coverage and thicker membranes [39].

Interestingly, even though a concentration of 3.75 mg/mL yielded poor results with the POPC/SM/Chol mixture, our earlier experiments using POPC/Chol binary mixtures were quite successful at that concentration [35]. A comparison of thicknesses of 2/1 POPC/Chol and 1/1/1 POPC/SM/Chol mixtures at 3.75 mg/mL showed a slight increase after addition of SM (33.2 ± 0.3 vs. 39 ± 1 nm, respectively, *p* = 0.009). A recent molecular simulation study found that introduction of SM to POPC/Chol bilayers increases hydrocarbon chain order, condenses the bilayer, and reduces water permeability [40]. The effect of SM is, thus, similar to the effect of Chol. Consequently, the decrease in electroformation successfulness when SM is added to the mixture can be explained through the effects of SM on bilayer properties and the difference in lipid film thickness.

In accordance with the results for different film thicknesses, the results for the 2.75 mg/mL concentration were superior to other combinations. The difference was most prominent at higher Chol concentrations, where different combinations showed very poor GUV quality. Moreover, after the Chol/(POPC + SM) mixing ratio reached 2.75, crystal-like patterns started appearing over the lipid-deposited surface. These probably represent Chol crystals forming due to the Chol demixing artefact (Figure 6b) [13,22,23]. Comparing the results between two different voltages for a lipid concentration of 3.75 mg/mL, we can see that using the optimal electrical parameters of 10 Hz–2 V as determined above did somewhat improve the quality of GUV preparations. However, successfulness for Chol/(POPC + SM) mixing ratios higher than 2 was still unsatisfactory, with low GUV yields and a large amount of defects. The 2.75 mg/mL concentration proved to be superior in this aspect as well, allowing the formation of a large number of GUVs with a much lower amount of defects. These findings show that optimizing the lipid film thickness is at least as important as optimizing the electrical parameters and should be performed for every new mixture used in order to maximize electroformation successfulness.

## 4. Conclusions

Combining spin-coating with the electroformation method, we investigated the effect of electrical parameters, film thickness, and Chol concentration on GUV production. The best frequency–voltage combinations were in the ranges 2–6 V and 10–100 Hz. Using AFM, we found the optimal film thickness to be approximately 30 nm. This thickness was achieved for a lipid solution concentration of 2.75 mg/mL at 600 rpm. Compared to binary POPC/Chol mixtures, SM seemed to make it harder for GUVs to form. Additionally, increasing the Chol concentration also led to lower-quality GUVs and a larger amount of defects. However, the effect was much less pronounced when the optimal film thickness was used during electroformation. At very high Chol concentrations (above a Chol/(POPC + SM) mixing ratio of 2.75), needle-like shapes started showing up on fluorescence microscopy images of lipid film surfaces. These most likely appeared due to Chol demixing during lipid film drying. Alternative protocols bypassing the dry lipid film state, such as those laid out by Baykal-Caglar et al. [23], could be applied to such mixtures. On the basis of their results for mixtures with lower Chol concentrations, we think that the protocol could be extended to concentrations exceeding the Chol saturation threshold.

In conclusion, the article presented the optimal values of several key electroformation parameters. The results confirm the need to optimize the protocol for every new mixture used and underlines the importance of lipid film thickness in the process.

## Data Availability

The data presented in this study are available upon reasonable request from the corresponding author.

## Supplementary Materials

The following supporting information can be downloaded at https://www.mdpi.com/article/10.3390/membranes12050525/s1: Figure S1: XRR analysis of spin-coated lipid films thicknesses. The right column shows the raw data, and the left one the normalized Fourier transform intensity. (a) Measurements for an ITO sample. (b) Measurements for different lipid concentrations using a Chol/(POPC+SM) mixing ratio of 0.75.
