# Supplementary material for: Optimization of Giant Unilamellar Vesicle Electroformation for Phosphatidylcholine/Sphingomyelin/Cholesterol Ternary Mixtures
Source: Membranes (Basel). 2022 May 16;12(5):525. doi: 10.3390/membranes12050525 (PMC9144572; doi:10.3390/membranes12050525)
Supplement: Supplementary file 1 [file membranes-12-00525-s001.zip › membranes-1692107-supplementary.pdf]

Supplementary Material

Optimization of Giant Unilamellar Vesicle Electroformation for Phosphatidylcholine/Sphingomyelin/Cholesterol Ternary Mixtures

Zvonimir Boban [1,2], Ivan Mardešić [1,2], Witold Karol Subczynski [3], Dražan Jozić [4] and Marija Raguz [1,*]

[1] Department of Medical Physics and Biophysics, University of Split School of Medicine, 21000 Split, Croatia; zvonimir.boban@mefst.hr (Z.B.); imardesi@mefst.hr (I.M.)
[2] Faculty of Science, University of Split, Doctoral Study of Biophysics, 21000 Split, Croatia
[3] Department of Biophysics, Medical College of Wisconsin, Milwaukee, WI 53226, USA; subczyn@mcw.edu
[4] Faculty of Chemistry and Technology, University of Split, 21000 Split, Croatia; jozicd@ktf-split.hr
* Correspondence: marija.raguz@mefst.hr; Tel.: +385-9876-8819

Figure S1 shows thicknesses obtained using XRR measurements. The right column shows the raw data, and the left one the normalized Fourier transform intensity. Curves for the ITO sample show a dominant peak centered at ~ 25 μm, with an additional smaller peak at ~ 55 μm (Figure S1a).

Figure S1b compares the thicknesses measurements between films made using three different lipid film concentrations and a Chol/(POPC+SM) mixing ratio of 0.75. The results for the 2 mg/ml concentration show an additional hump around 20 μm. This is the dominant peak when we account the ITO signal and its value is in agreement with the AFM measurements. The middle concentration mostly restores the symmetrical shape of the main ITO peak, with a dominant lipid film peak occuring at ~ 35 μm. The highest measured concentration seems to be the least homogenous, but an increase in thickness compared to lower concentrations is evident from the wide peak appearing at ~ 70 μm. The results are in line with the trend of increasing thickness with increasing lipid concentration suggested by the AFM results in the main text.

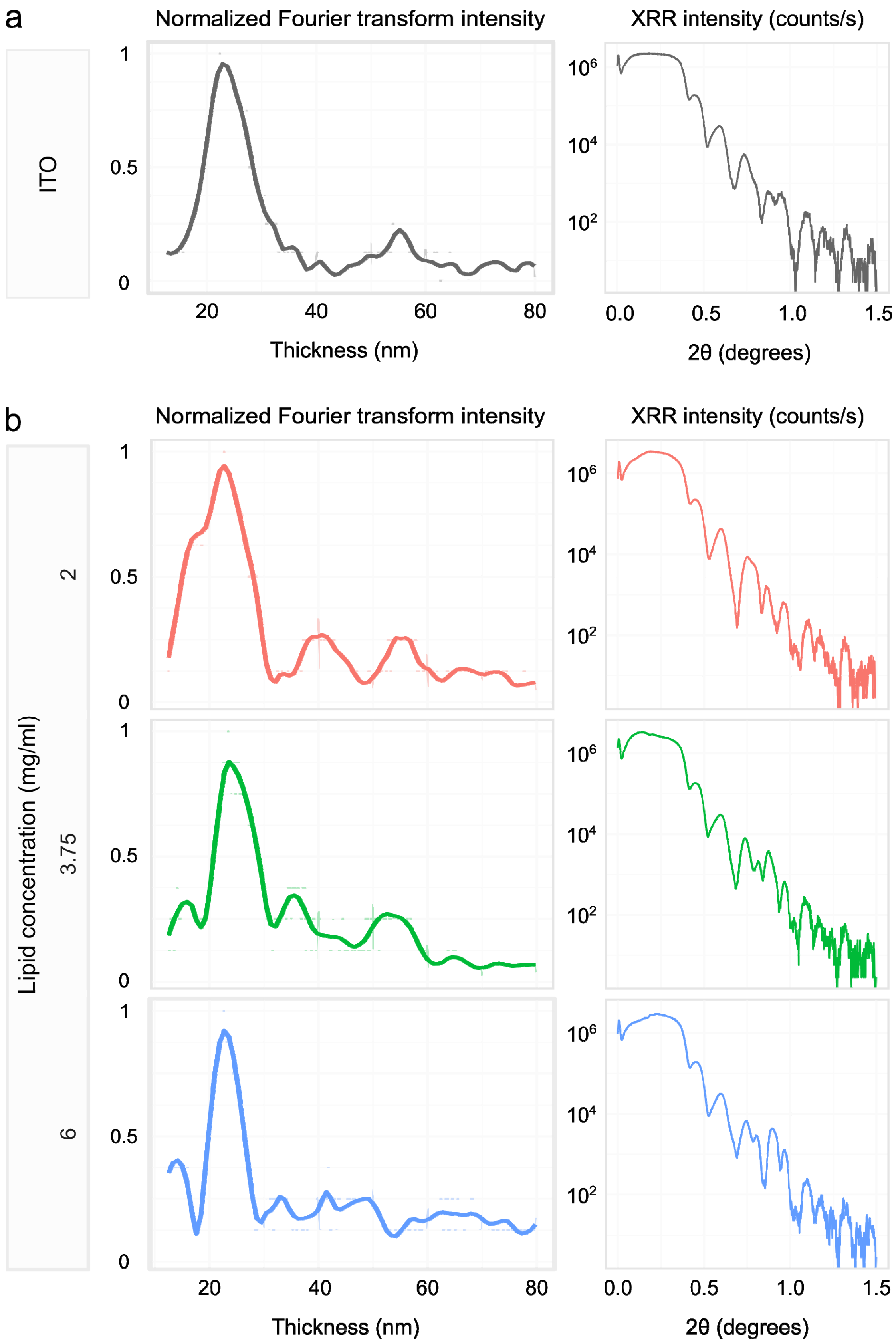

Figure S1. XRR analysis of spin-coated lipid films thicknesses. The right column shows the raw data, and the left one the normalized Fourier transform intensity. **(a)** Measurements for an ITO sample. **(b)** Measurements for different lipid concentrations using a Chol/(POPC+SM) mixing ratio of 0.75.
